# An Ethanolic Extract of* Allium hookeri* Root Alleviates Reflux Esophagitis and Modulates NF-*κ*B Signaling

**DOI:** 10.1155/2018/1834681

**Published:** 2018-10-08

**Authors:** Li Nan, Hyeon Hwa Nam, Byung Kil Choo, Jin Cheon Park, Dae Geun Kim, Jeong Ho Lee, Kwang Hyun Moon

**Affiliations:** ^1^Department of Crop Science & Biotechnology, Chonbuk National University, Jeonju 561-756, Republic of Korea; ^2^Sunchang Research Institute of Health and Longevity, Ingye-myeon Indeok-ro, Sunchang-gun, Jeollabuk-do 56015, Republic of Korea

## Abstract

Reflux esophagitis (RE) is a kind of gastroesophageal reflux disease, of which an esophageal inflammatory lesion is caused by the contents of the stomach and duodenum flowing back into the esophagus.* Allium hookeri* is a plant possessing both nutritional and medicinal properties. In our study, we investigated the inhibition effect of inflammation of* A. hookeri* root extract (AHE) on inflammatory RAW264.7 macrophage cells induced by lipopolysaccharide and rat models of RE. The results showed that AHE significantly reduced the production of nitric oxide (NO) and the protein expression levels of various mediators related to inflammation including inducible nitric oxide synthase (iNOS), cyclooxygenase-2 (COX-2), and inflammatory cytokines such as interleukin-1 beta (IL-1*β*) and tumor necrosis factor-alpha (TNF-*α*). Furthermore, AHE also inhibited the nuclear translocation of nuclear factor kappa B (NF-*κ*B) by inhibiting the phosphorylation I*κ*B*α*. In addition, AHE administration significantly ameliorated esophageal mucosal damage upon histological evaluation of RE in rats. AHE was also found to downregulate the expression levels of proteins such as COX-2, TNF-*α*, and IL-1*β* in the rat esophagus. AHE markedly attenuated activation of NF-*κ*B and phosphorylation of I*κ*B*α* at the same time. These results indicated that AHE suppressed LPS-induced inflammatory responses in RAW264.7 cells and may help reduce the development of esophagitis through the modulation of inflammation by regulating NF-*κ*B activation.

## 1. Introduction

Gastroesophageal reflux disease (GERD) is a disorder with prevalence up to 20-40% in America and Europe [1]. The prevalence of GERD is also increasing in the Asian areas [2]. GERD is a condition in which stomach contents which frequently flow back into esophagus cause troublesome symptoms and/or complications [3]. The main features of GERD include heartburn and acid regurgitation [4].

Reflux esophagitis (RE) is a kind of gastroesophageal reflux disease, which is an esophageal inflammatory lesion caused by the contents of the stomach and duodenum flowing back into the esophagus, that is, esophageal erosion or esophageal ulcer [5]. The main cause of RE is the destruction of the reflux barrier (lower esophageal sphincter). The LES is a high-pressure area within 3-5 cm above the junction between the esophagus and the stomach. It plays a physiological role in preventing the flow of stomach contents into the esophagus [6]. Symptoms such as erosion and stenosis in the lower esophagus are considered to be typical complications of RE [7].

Inflammation is a defensive reaction to biological threats. However, persistent or prolonged inflammation may lead to the development of extensive tissue damage or disease, including cancer. The use of plant-based medicinal compounds for the reduction of inflammation has become a popular topic in recent years. In one study, paeonol, the main component of Moutan Cortex, has been shown to exhibit anti-inflammatory effects by decreasing the level of proinflammatory mediators [8]. In addition, ethanol extracts of* Sanguisorba officinalis* L. have been observed to inhibit both the degradation of I*κ*B*α* and the nuclear translocation of NF-*κ*B p65, confirming its anti-inflammatory capacity [9].

Lipopolysaccharide is a component of the cell wall of Gram-negative bacteria and is toxic to the host [10]. It is always used to induce inflammatory responses in various experimental models [11]. Macrophages stimulated by LPS produce many proinflammatory factors and inflammatory mediators such as NO, iNOS, COX-2, TNF-*α*, and IL-1*β* that regulated by NF-*κ*B [12, 13]. Under homeostatic conditions, NF-*κ*B binds to its inhibitory protein (I*κ*B) in the cytoplasm, assuming an inactive state. Stimuli such as LPS, TNF-*α*, and IFN-*γ* can induce the phosphorylation of I*κ*B and resulting activation of NF-*κ*B. The activated NF-*κ*B is transferred to the nucleus and binds to a specific DNA target site to regulate transcription of the target gene [14].

*Allium hookeri* (also known as Hooker chives or Kuan ye jiu) is a perennial herbaceous evergreen plant, distributed in Yunnan, Sichuan, and Southeastern China, as well as in Sri Lanka, Bhutan, and India [15, 16]. The nutritional and medicinal properties of* A. hookeri* have been described previously. As a supplementary food, it contains various nutrients including sugars, phenol, phytosterols, vitamin C, fiber, and protein [17, 18]. Some studies reported that these nutrients are present in higher quantities in* A. hookeri* than in* Allium cepa* [12, 19]. Alliin, a garlic organosulfur compound, has been reported to inhibit LPS-induced inflammatory response [20, 21] and protect against LPS-induced acute lung injury [22]; the compound has been proven to be present in* A. hookeri* [23]. Meanwhile, the various medicinal effects of* A. hookeri* have been demonstrated to include anticoagulant [24], antidiabetic [25], anticholesterol [26], antibacterial [27], and antiobesity [28] activities. The root extract has also been shown to be beneficial for bone health and can lower blood glucose levels while increasing insulin sensitivity [29].

Although* A. hookeri* has been shown to have anti-inflammatory effects, whether these effects extend to the inhibition of elements of RE is not well known. In this study, we investigated the therapeutic potential of* A. hookeri* root extract (AHE) in LPS-induced damage in the RAW264.7 macrophage cell line. Furthermore, we investigated the effect of this extract on rat models of RE to explore the possible underlying mechanisms of inhibition.

## 2. Materials and Methods

### 2.1. Materials

The protease inhibitor cocktail, PMSF, and EDTA were obtained from Sigma Aldrich (Seoul, Korea). LPS was purchased from Sigma Chemical Company. The Cell Viability, Proliferation & Cytotoxicity Assay Kit was purchased from DoGenBio Co., Ltd. (Seoul, Korea). The bovine serum albumin standard, protein assay reagent, and PVDF were purchased from Bio-Rad Laboratories (Hercules, CA, USA). Griess reagent was obtained from Promega (Madison WI, USA). iNOS, COX-2, *β*-actin, p-I*κ*B*α*, p-NF-*κ*B p65, IL-1*β*, and TNF-*α* antibodies and Luminol Reagent were purchased from Santa Cruz Biotechnology, Inc. (Santa Cruz, CA, USA).

### 2.2. Preparation of AHE

*A. hookeri* roots were purchased from Sunchon-myeon, Jeollabuk-do, Korea. After the roots are dried, they are ground into a powder. The powder was suspended in ten volumes of 75 % ethanol and extracted for 2 h four times under a 50°C circulation distillation apparatus. The ethanol extract was filtered, concentrated, lyophilized, and then stored at -80°C until used.

### 2.3. Cell Culture

Macrophage of RAW264.7 cells was obtained from ATCC (Rockville, MD, USA). Cells were cultured in DMEM containing 10 % heat-inactivated FBS (Welgene, Namcheon-ro, South Korea), 100 units/ml penicillin, and 100 *μ*g/ml streptomycin. The cells are grown in a constant temperature incubator with a CO_2_ of 5% and a temperature of 37°C.

### 2.4. Cell Viability and NO Assay

To determine cell viability, cells were distributed at a concentration of 5×10^5^cells/ml on a 96-well cell culture plate. They were then treated with AHE (125, 250, and 500 *μ*g/ml). After 1h, LPS (1 *μ*g/ml) was added to the plate for an additional 24 h. Viability of cells was determined by the Cell Viability, Proliferation & Cytotoxicity Assay Kit. Determination of nitrite content in culture medium was using Griess reagent (Promega, Madison, WI, USA). The NO concentration was determined by measuring the absorption at 540 nm using a NaNO_2_ dilution set for a standard curve. Each treatment was carried out in triplicate.

### 2.5. Cell Protein Extraction

In order to extract the total protein of the cells, the cells were plated at a concentration of 1×10^6^ cells/ml in a 60×15 mm cell culture dish and treated with AHE (250, and 500 *μ*g/ml) for 1h. LPS (1 *μ*g/ml) was then added to the plate, and the cells were incubated for 1 h or 18 h. Cells were washed with PBS 3 times and then centrifuged at 4,000 rpm for 3 min. The final pellet was lysed in 100 *μ*l RIPA lysis buffer with 150 mM NaC1, 5 mM EDTA (pH 8.0), 50 mM Tris (pH 8.0), 1 % NP-40, 0.1 % sodium dodecyl sulfate, 0.5 % sodium deoxycholate, and protease inhibitor mixture solution on ice for 15 min. The lysis mixture was then centrifuged at 13,000 rpm for 15 min, and the supernatant was taken up in EP tube and stored at -80°C until used.

### 2.6. Experimental Animals and Treatment

Sprague Dawley rats (7-week-old, body weight 200-220g) were housed in standard rat cages for experimentation, providing adequate food and water at all times, maintained in a 12-hour light/dark cycle, at a temperature of 21-25°C and a humidity of 35-60%. After one week of adaptation, the rats were randomly divided into 3 groups of 6 each which were normal group, RE control group, and drug treatment RE group. The rats were fasted 18h before the operation but maintained water supply. Rats in the RE control group and the drug-treated RE group were intragastrically administered 1.5 h before the operation of inducing reflux esophagitis. The RE control group was physiological saline, and the drug treatment group was AHE at a concentration of 500 mg/kg. Then the rats were subjected to respiratory anesthesia, and an incision of about 2 cm was cut in the middle part of the abdomen of the rat to expose the stomach, and then the stomach and pylorus junctions and the fundus were ligated to induce reflux, keeping the vagus nerve intact [30]. After 4.5 h of surgery, all rats were sacrificed. The esophagus was immediately removed and washed with saline, photographed (for the calculation of the degree of esophageal damage), and then the esophageal tissue was stored at -80°C for later used.

### 2.7. Esophageal Injury Ratio

The esophagus of the rat was cut longitudinally, and the damaged part was exposed. After the photography, the image of the esophageal injury rate was calculated by ImageJ software [31]. The calculation method of the esophageal injury ratio is as follows: gross mucosal damage ratio (%) = [area of esophageal mucosal damage (mm^2^)/total area of esophagus (mm^2^)] × 100.

### 2.8. Esophageal Histology Analysis

After the rat esophagus was removed, it was washed with physiological saline, then cut into small pieces of 2-3 mm, and immersed in 10% of neutral buffered formalin. The esophageal specimens were rinsed, dehydrated, transparent, dipped in wax, embedded, sectioned (5 *μ*m), and finally stained with hematoxylin and eosin and fixed on a slide. Collect the digital images using a Leica microscope (magnification was 100 x).

### 2.9. Extraction of Nuclear and Cytoplasmic Protein Components from Esophageal Tissues

The protein extraction process of the cells was carried out in accordance with the method described by Komatsu and colleagues [32]. Briefly, the esophageal tissue was weighed and homogenized in tissue lysis buffer contained with protease inhibitor cocktail, 10 mM HEPES (pH 7.8), 10 mM KCl, 2 mM MgCl_2_, 0.1 mM EDTA, 0.1mM PMSF, and 1 mM DTT. Then the lysate was placed on ice for 30 min and mixed once every 10 min. The lysate was centrifuged at 13,000 rpm for 3 min at 4°C, and the supernatant containing cytoplasmic protein component was collected. The bottom pellet was resuspended with lysis buffer with protease inhibitor cocktail, 50 mM HEPES (pH 7.8), 50 mM KCl, 300 mM NaCl, 1 mM DTT, 0.1 mM EDTA, 0.1mM PMSF, and 1% glycerol. The lysate were placed on ice for 30 min, mixed once every 10 min, and centrifuged at 13,000 rpm for 15 min at 4°C. Finally, collect supernatant containing nuclear protein components and store at -80°C until used.

### 2.10. Western Blot Analysis

The loading sample was separated by SDS-PAGE, transferred to a PVDF membrane and then blocked with 5% skim milk for 1 h 30 min at room temperature. Membranes were incubated with primary antibodies (1:1,000) against iNOS, COX-2, TNF-*α*, IL-1*β*, p-NF-*κ*B p65, p-I*κ*B*α*, histone, and *β*-actin at 4°C overnight. Then, the secondary antibody was added to react at room temperature for 1.5 h with gentle agitation. Bands were visualized using western blotting Luminol Reagent solutions A and B at a 1:1 proportion. Images were acquired with Bio-Rad imaging software (Fuji, New York, NY, USA).

### 2.11. Statistical Analysis

All data results are mean ± standard deviation. Significant evaluation was performed by one-way analysis of variance and LDS's multiple comparison test, statistically significant at p<0.05.

## 3. Results

### 3.1. AHE Treatment Does Not Affect Cell Viability

**Figure 1:**
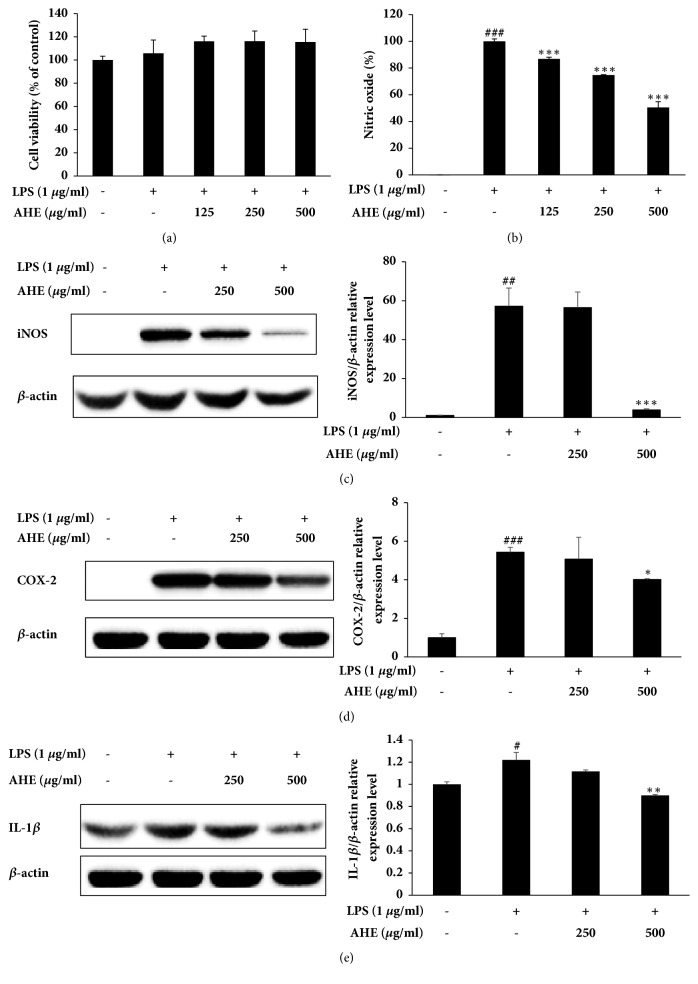
Safety and anti-inflammatory activities of AHE in LPS-induced RAW264.7 cells. The cells were treated with AHE at various concentrations for 1 h and then stimulated, or not, with LPS for 24 h or 18 h. Cell viability (a) was assayed by the Cell Viability, Proliferation & Cytotoxicity Assay Kit, and the production of NO (b) was measured using the Griess test. The expression levels of iNOS (c), COX-2 (d), and IL-1*β* (e) in LPS-induced RAW264.7 cells were measured using western blot assay. Values are presented as the mean ± standard deviation of three independent experiments. ^###^P<0.001, ^##^P<0.01, and ^#^P<0.05 versus unstimulated cells; ^*∗∗∗*^P<0.001, ^*∗∗*^P<0.01, and ^*∗*^P<0.05 versus LPS-stimulated cells.

The effect of AHE on the viability of RAW264.7 cells was evaluated using the Cell Viability, Proliferation & Cytotoxicity Assay Kit. No significant effects on cell viability were detected following 24 h incubation with AHE at concentrations of 125, 250, and 500 *μ*g/ml (Figure 1(a)).

### 3.2. AHE Treatment Inhibits the Production of LPS-Induced NO and iNOS

As shown in Figure 1(b), the production of NO was inhibited by treatment with AHE in a concentration-dependent manner. The expression level of iNOS, a protein regulated by NF-*κ*B activity, was also reduced in a dose-dependent manner (Figure 1(c)).

### 3.3. AHE Treatment Inhibits LPS-Induced Production of COX-2 and IL-1*β*

To investigate the expression levels of COX-2 and IL-1*β*, cells were incubated with varying concentrations of AHE for 1 h prior to stimulation with LPS (1 *μ*g/ml) for 18 h. As shown in Figure 1(d), AHE inhibited COX-2 expression in a concentration-dependent manner. In addition, AHE suppressed the expression level of the proinflammatory cytokine IL-1*β* (Figure 1(e)).

### 3.4. AHE Treatment Inhibits LPS-Induced NF-*κ*B Activation

**Figure 2:**
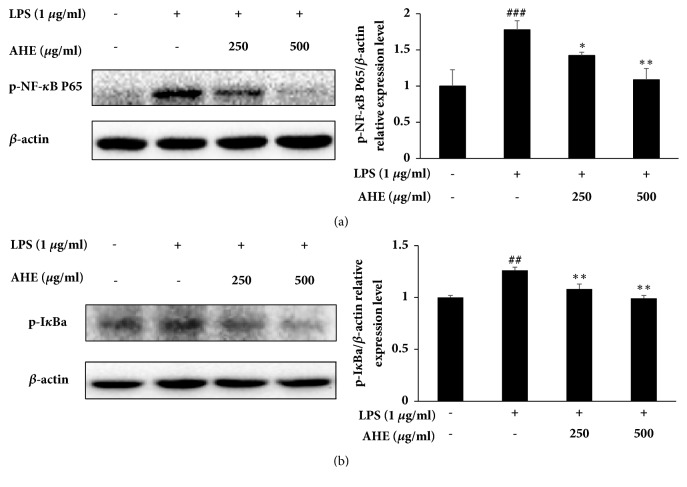
The signaling pathway involved in the anti-inflammatory activities of AHE in LPS-induced RAW264.7 cells. The expression levels of p-NF-*κ*B (a) and p-I*κ*B*α* (b) in LPS-induced RAW264.7 cells were measured using western blot assay. Values are presented as the mean ± standard deviation of three independent experiments. ^###^P<0.001, ^##^P<0.01, and ^#^P<0.05 versus unstimulated cells; ^*∗∗*^P<0.01 and ^*∗*^P<0.05 versus LPS-stimulated cells.

To investigate whether AHE treatment regulates the NF-*κ*B signaling pathway, cells were treated with different concentrations of AHE for 1 h and then stimulated with LPS (1 *μ*g/ml) for another 1 h. As shown in Figure 2(a), AHE significantly inhibited the expression of phosphorylated NF-*κ*B. The expression level of phosphorylated I*κ*B*α* was also inhibited after AHE treatment (Figure 2(b)).

### 3.5. AHE Treatment Attenuates Gross Mucosal Damage of the Esophagus in RE Rat

**Figure 3:**
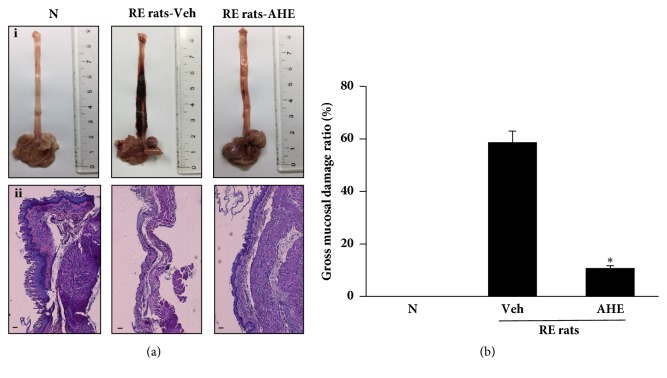
Effect of AHE on RE rats. Morphological ((a)-i) and histological ((a)-ii) examination of the esophagus in each group. Gross mucosal damage ratio (b). N, normal rats; Veh, RE control rats; AHE, RE control rats treated with 500 mg/kg AHE (scale bar 200 *μ*m). Values are presented as the mean ± standard deviation. ^*∗*^P<0.05 versus RE control rats.

As shown on Figure 3(a)-i, in the normal group, no damage of rat esophagus was observed, but in the RE control group, the rat's esophagus showed severe damage, including congestion and multiple erosions. In the AHE treatment group, it can be seen that the esophageal injury is significantly reduced. As shown on Figure 3(b), gross mucosal damage ratio in AHE group was significantly lower than that in RE control group. In addition, it can be seen from histological staining that the normal mucosal epithelium was observed in the normal group and showed a thin epithelial layer, and there was no infiltration of inflammatory cells in the submucosa. Conversely, significant tissue damage can be observed in the RE control group, showing mucosal damage to the esophageal tissue, massive loss of epithelial cells, marked epithelial hyperemia, and mucosal and submucosal hemorrhage, while significantly less damage to the esophageal tissue was seen in mice that had received oral AHE (500 mg/kg) (Figure 3(a)-ii).

### 3.6. AHE Treatment Reduces the Expression of Proinflammatory Mediators in the RE Rat Esophagus

**Figure 4:**
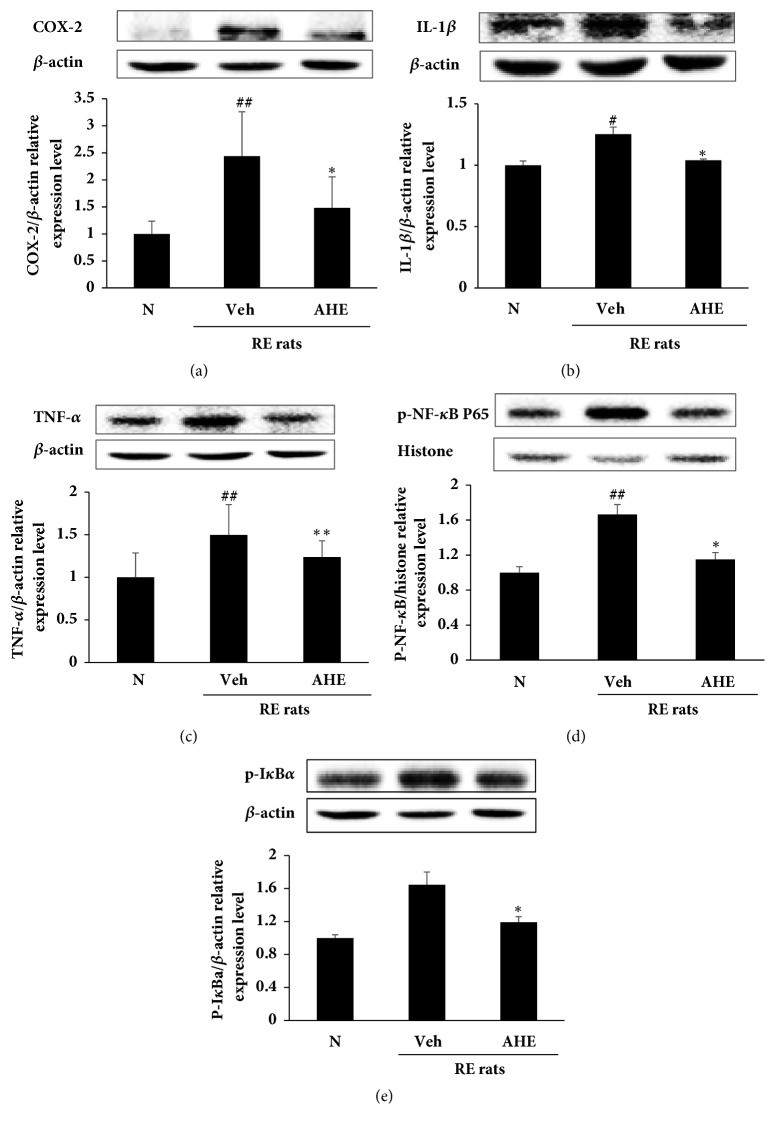
Effects of AHE on the expression levels of COX-2 (a), IL-1*β* (b), and TNF-*α* (c) and the phosphorylation levels of NF-*κ*B (d) and I*κ*B*α* (e) in rat esophageal tissue were measured using western blot assay. N, normal rats; Veh, RE control rats; AHE, RE control rats treated with 500 mg/kg AHE. ^##^P<0.01 and ^#^P<0.05 versus normal rats; ^*∗∗*^P<0.01 and ^*∗*^P<0.05 versus RE control rats.

As shown in Figure 4, the expression levels of mediators related to inflammation and inflammatory cytokines, including COX-2 (a), IL-1*β* (b), and TNF-*α* (c), were reduced in AHE-treated RE rats when compared with RE control rats. In addition, the expression levels of p-NF-*κ*B (d) and p-I*κ*B*α* (e) were increased in the esophagus tissue of RE rats, while these levels were markedly decreased in the AHE-treated RE rats.

## 4. Discussion

In this study, we first observed the inhibitory effect of AHE on LPS-induced inflammation. We found that AHE treatment of LPS-induced RAW264.7 cells markedly inhibited the production of NO, mediators related to inflammation such as iNOS and COX-2, and cytokines such as IL-1*β* and TNF-*α*. In addition, AHE decreased the phosphorylation of NF-*κ*B and p-I*κ*B*α*. We then investigated whether AHE has protective effects against the development of experimental rat RE. The administration of AHE was found to alleviate the degree of esophageal tissue mucosal damage and the protein expression levels of inflammatory mediators and cytokines associated with RE.

Inflammation is an innate immune response of the host to foreign aggressions such as pathogens, bacteria, and tissue damage [33]. The inflammatory response occurs in many types of cells, including macrophages and monocytes. When the inflammatory reaction occurs, the body mainly manifests as redness, fever, swelling, and dysfunction [34]. RE is a common gastroesophageal reflux disease that is common in Europe and other areas that seriously affects the quality of life of human beings. In recent years, there has also been a gradual upward trend in the Asian region [35, 36]. Persistent esophageal reflux can lead to esophageal inflammation, and prolonged esophagitis is likely to induce esophageal cancer.

The NF-*κ*B transcription factor comprises five dimeric complexes such as p65 (RelA), c-Rel, RelB, p50, and p52. Each of these dimeric complexes has a 300-residue N-terminal Rel-homologous domain, which is primarily responsible for dimerization, nuclear transfer, and DNA binding. [37, 38]. The NF-*κ*B signaling pathway is thought to be a classical signal transduction pathway regulating inflammatory responses. NF-*κ*B is activated by stimulants such as LPS and separated from its inhibitory protein I*κ*B and transferred into the nucleus to promote gene expression of inflammation-related factors [39]. In resting cells, NF-*κ*B is mainly present in the cytoplasm, and I*κ*B proteins inhibit their activity by masking their nuclear localization sequences (NLSs). Excessive activation of NF-*κ*B may lead to the development of a variety of inflammatory diseases and even cancer [40]. Activated NF-*κ*B enters the nucleus to bind to DNA and induce the transcription of target genes such as TNF-*α* and IL-1*β*. It also regulates the expression of inducible enzymes such as iNOS and COX-2.

In this study, AHE treatment showed promising effects against LPS-induced inflammation in RAW264.7 cells. Furthermore, AHE treatment of the RE rats obviously inhibited upregulation of inflammatory mediators related to NF-*κ*B signaling in esophageal tissue. In addition, the expression levels of TNF-*α* and IL-1*β* in the RE model were significantly downregulated by the administration of AHE.

## 5. Conclusions

In conclusion, we demonstrated that AHE inhibits LPS-induced inflammation of macrophage RAW 264.7 cells by inhibiting NF-*κ*B activation. At the same time, we also found that treatment with AHE can effectively improve the damage of the esophageal mucosa in the rat model of RE and inhibit the expression of inflammatory mediators and cytokines regulated by NF-*κ*B. In summary, our data extend the existing potential of medicinal use of* A. hookeri*.

## Data Availability

The data used to support the findings of this study are available from the corresponding author upon request.

## Abbreviations

AHE: * Allium hookeri* root extract
GERD: Gastroesophageal reflux disease
RE: Reflux esophagitis
LPS: Lipopolysaccharide
NO: Nitric oxide
iNOS: Inducible nitric oxide synthase
COX-2: Cyclooxygenase-2
IL-1*β*: Interleukin-1*β*
TNF-*α*: Tumor necrosis factor- *α*
NF-*κ*B: Nuclear factor *κ*B
I*κ*B*α*: Nuclear factor of kappa light polypeptide gene enhancer in B-cells inhibitor-*α*
EDTA: Ethylenediaminetetraacetic acid
H&E: Hematoxylin and eosin
HEPES: 4-(2-Hydroxyethyl)-1-piperazineethanesulfonic acid
SDS: Sodium dodecyl sulfate
DTT: DL-Dithiothreitol
PMSF: Phenylmethylsulfonyl fluoride
EDTA: Ethylenediaminetetraacetic acid
PVDF: Polyvinylidene fluoride membranes
ATCC: American Type Culture Collection.
